# Reply to Dey and Schreiber: Porous media framework of intracellular diffusion is not limited to inert molecules

**DOI:** 10.1073/pnas.2606660123

**Published:** 2026-04-10

**Authors:** Olivier Destrian, René-Marc Mège, Benoît Ladoux, Benoît Goyeau, Morgan Chabanon

**Affiliations:** ^a^Université Paris-Saclay, CNRS, CentraleSupélec, Laboratoire d’Energétique Moleculaire et Macroscopique, Combustion (EM2C), Gif-sur-Yvette 91190, France; ^b^Université Paris-Cité, CNRS, Institut Jacques Monod, Paris 75013, France; ^c^Department of Physics, Friedrich-Alexander Universität Erlangen-Nürnberg, Erlangen 91058, Germany; ^d^Max-Planck-Zentrum für Physik und Medizin and Max Planck Institute for the Science of Light, Erlangen 91054, Germany

In their letter, Dey and Schreiber (1) recognize the relevance of the porous-media framework presented in Destrian et al. (2) to study intracellular diffusion of inert macromolecules. However, they also highlight that the cytoplasm and many biomolecules are charged, requiring us to generalize our model to electrostatic interactions. While we agree with this analysis, here we argue that a large part of the data used by Dey and Schreiber supports that the diffusive properties of weakly negatively charged molecules are assimilable to inert molecules, confirming the relevance of our approach. Additionally, we highlight that one of the strengths of our model is its multiscale framework. This makes it readily suitable to be extended to more complex biophysical effects, such as electrostatic interactions.

**Fig. 1.**
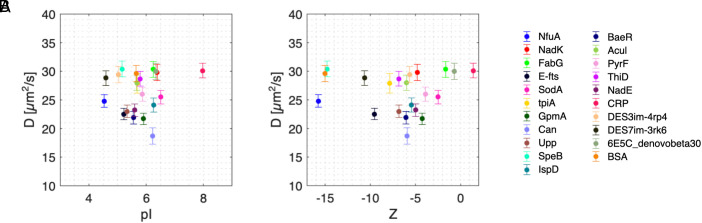
Experimental diffusion coefficient of negatively charged and neutral small bacterial proteins does not correlate with molecule isoelectric point nor net charge. Experimental diffusion coefficient as a function of (*A*) the isoelectric point (pA) and (*B*) of the particle net charge. All data are from ref. 4.

The experimental basis for the development of our multiscale model for cytoplasmic diffusion relies primarily on the use of green fluorescent protein (GFP) (figures 1 to 5 of ref. 2) that we approximate as inert (*SI Appendix*, section 5 of ref. 2), as well as inert Dextrans and Ficolls (figure 6 of ref. 2). We show that cytoplasm crowding induces both tortuous and porous hydrodynamic hindrances, significantly reducing diffusion of macromolecules. Though we believe that studying this drag term is a key finding as it universally applies to all macromolecules regardless of their chemical properties, we do not claim that other forces such as nonspecific electrostatic interactions are negligible for all molecules and cytoplasmic regions (3).

However, because the cytoplasm is slightly negatively charged, many proteins that are weakly negatively charged can be approximated as inert. The data presented in ref. 1, actually supports this claim by showing that, for small bacterial proteins microinjected inside Hela cells (4), the diffusion coefficient of neutral or weakly negatively charged particles of comparable molecular weights is uncorrelated with their charge (Fig. 1). This suggests that electrostatic interactions play a minor role on the cytoplasmic mobility of molecules of biological interest that are neutral or weakly negatively charged, as supported by others (5, 6).

Yet, for positively charged molecules or small molecules, nonspecific electrostatic interactions are expected to dominate over steric effects. The model we established for inert particles (2) can be readily expanded building on the advances in the field of transfers in porous media. Upscaling electrodiffusive transport in charged microstructures has been largely studied within our methodological framework (7, 8). It requires modifying at the pore scale the diffusive term to include Nernst–Planck equation and to account for diffusive double layers at the structure interfaces. Interestingly, the upscaling procedure indicates that electrostatic interactions reduce to a reaction-like term in the macroscopic transport equation, instead of an effective diffusion coefficient (7–9). This has direct consequences on the interpretation of experimental fitting procedures to determine experimentally the diffusion coefficient in live cells.

We thank Dey and Schreiber for this opportunity to discuss the domain of validity and possible extensions of our porous-media framework for intracellular diffusion in the context of electrostatic and nonspecific interactions.
